# Understanding the Intergenerational Impact of Migration: An Adult Mortality Advantage for the Children of Forced Migrants?

**DOI:** 10.1097/EDE.0000000000001763

**Published:** 2024-07-10

**Authors:** Ben Wilson, Matthew Wallace, Jan Saarela

**Affiliations:** From the a Department of Sociology, University of Stockholm, Stockholm, Sweden; b Department of Methodology, London School of Economics, London, United Kingdom; c Demography Unit, Åbo Akademi University, Vaasa, Finland.

**Keywords:** Adult mortality, Cause of death, Children of immigrants, Finland, Forced migration, Second generation

## Abstract

**Background:**

Children of immigrants often have excess mortality rates, in contrast to the low mortality typically exhibited by their parents’ generation. However, prior research has studied children of immigrants who were selected for migration, thereby rendering it difficult to isolate the intergenerational impact of migration on adult mortality.

**Methods:**

We use semiparametric survival analysis to carry out a total population cohort study estimating all-cause and cause-specific mortality among all adult men and women from age of 17 years among all men and women born in 1953–1972 and resident in Finland in 1970–2020. We compare children of forced migrants from ceded Karelia, an area of Finland that was ceded to Russia during the Second World War, with the children of parents born in present-day Finland.

**Results:**

Children with two parents who were forced migrants have higher mortality than children with two parents born in Northern, Southern, and Western Finland, but similar or lower mortality than the subpopulation of children whose parents were born in the more comparable areas of Eastern Finland. For women and men, a mortality advantage is largest for external causes and persists after controlling for socioeconomic factors.

**Conclusion:**

Our findings suggest that forced migration can have a beneficial impact on the mortality of later generations, at least in the case where forced migrants are able to move to contextually similar locations that offer opportunities for rapid integration and social mobility. The findings also highlight the importance of making appropriate comparisons when evaluating the impact of forced migration.

Many high-income societies have witnessed considerable growth in the share of the resident population who are second-generation [G2] children of immigrants, defined here as native-born individuals with at least one foreign-born parent.^1^ The average share of the adult G2 across the European Union is 7%, but shares are much larger in countries such as Belgium (13%), France (13%), and Sweden (11%).^2^ With respect to mortality, research has shown that G2 children of immigrants often exhibit elevated mortality compared with children of two native-born parents (i.e., the ancestral native-born population).^3–12^ This stands in contrast with the lower mortality typically exhibited by their parents, the first generation (G1).^13,14^

Despite what we know about mortality for G2 children of immigrants, prior research is based on studies of children born to immigrants who were selected for migration.^4,5^ This makes it hard to isolate the intergenerational impact of migration on adult mortality for G2 because it is likely to be confounded by selection into migration of the G1, more specifically factors relating to this selection process that have an impact on their G2 children.^3,5,13,15^ Here we address this issue by investigating a unique case of forced migration that effectively eliminates selection as a determinant of the mortality of G1 and G2.

In addition to the role of selection, migration scholars theorize that G1 immigrants experience an ongoing process of adaptation after arrival that encompasses factors such as culture, language, and discrimination.^16^ They also theorize that this process is intergenerational, impacting the G2 directly, as well as indirectly via their parents’ adaptation.^15,16^ Adaptation is therefore likely to have an impact on the mortality of G2 children of immigrants. However, given our interest in the intergenerational impact of their parents’ migration, which occurs prior to any adaptation of G1 or G2, we conceptualize adaptation as a mechanism (and mediator) in the research that follows.

Forced migrants are typically defined by migration scholars as refugees, asylum seekers, and those who are internally displaced, although the classification of forced migrants is complex because there are many reasons why migrants are forced to move, including conflicts, development projects, and natural disasters, which in some cases may be unknown.^17–20^ The determinants of forced migrants’ health and mortality are a topic of interest in its own right, not least as a guide to policymakers.^17,21^ In addition, forced migration is of interest to migration scholars because it is unanticipated and involves different processes of selection into migration as compared with other forms of migration.^17,19–21^

Here, we study a unique example of forced migration. Specifically, we exploit the forced displacement of an entire population during the Second World War, from ceded Karelia, an area in Northern Europe of historical significance for Finland and Russia, to present-day Finland.^22–24^ We study the children of those immigrants who were forced to migrate. Crucial to the design of this study, the circumstances surrounding forced migration from ceded Karelia means that individuals were not selected on observed or unobserved characteristics.^25^ Furthermore, there were negligible differences between ceded Karelia and the rest of Finland in terms of culture, language, and society. Nevertheless, it is true that forced migrants from ceded Karelia could be considered more similar to the population of eastern Finland, particularly those people closest to the border shared with ceded Karelia.^24^

We are not aware of prior research on the mortality of children of forced migrants who were born after their parents were forced to migrate.^17,19^ In general, research has been limited by a paucity of data, notably data with adequate statistical power to be able to analyze mortality.^26^ Among the G1, evidence suggests that forced migration can have a negative impact on all-cause mortality for migrants arriving as adults,^27^ but a negligible impact for migrants arriving as children.^28^ With respect to forced migrants from ceded Karelia, prior research shows that all-cause and cause-specific mortality among the G1 is higher than the population of present-day Finland.^22,23^ Yet, this negative mortality differential disappears when forced migrants are instead compared to the populations of eastern Finland, close to the border with ceded Karelia.^23,24^

Here, we aim to establish whether the children of Karelian forced migrants experience elevated mortality compared with a population of present-day Finland whose parents were not forced to migrate. By studying the children of forced migrants from Karelia, it is possible to establish whether excess mortality exists for a G2 population whose parents were not a selective subgroup of the origin population. In doing so, we are not only able to make conclusions about the intergenerational impact of forced migration but also to draw inferences about the role of selection in determining G2 mortality more generally. We return to this in the discussion, where we also discuss the potential generalizability of our findings, in particular given the likely role of adaptation and other mechanisms that may impact health and mortality in the context that we study.

## METHOD

### Study Context

**FIGURE 1.**
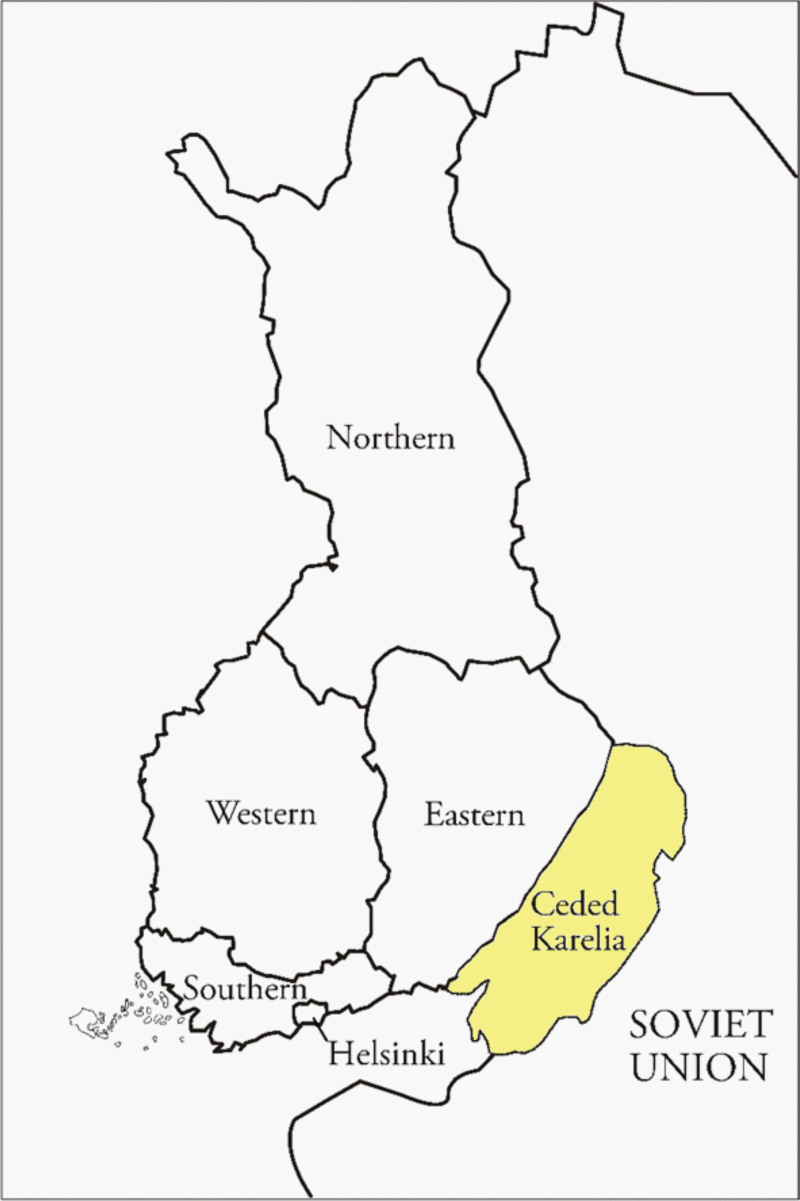
Map of Finland. This map shows present-day Finland separated into the regions of Northern Finland, Western Finland, Eastern Finland, Southern Finland, and the Helsinki metropolitan area. The area that was formerly part of Finland and ceded to the (former) Soviet Union after the Winter War of 1939–1940 is marked on the map as “Ceded Karelia.” A large part of this area is Lake Ladoga (not shown here but see eFigure S2; http://links.lww.com/EDE/C159). A much smaller part of Northern Finland was also ceded to the Soviet Union, but this is excluded from our study. As noted in the text, the supplementary materials include spatial information regarding our analysis of the border regions between Eastern Finland and ceded Karelia.

The Soviet annexation of Finnish Karelia is described in detail elsewhere,^22–24^ but can be summarized succinctly as follows. In a peace treaty of March 1940, Finland ceded roughly a tenth of its territory to the Soviet Union. The majority of this was in the south-east of Finland, as shown in Figure 1. Approximately 407,000 people, the entire population of these areas, were evacuated to the rest of the country in the spring and summer 1940.^29^ In June 1941, the ceded areas were then reoccupied by Finland and, from the end of 1941, two-thirds of those who had been displaced returned to their prewar homes. However, in the summer of 1944, the entire population of the ceded areas was again forced to relocate to present-day Finland, with no opportunity to return ever since.

### Study Design

We focus on children born in present-day Finland who have two parents born in ceded Karelia. In line with prior research, we assume that these are all the children of forced migrants, as justified by minimal levels of internal migration from ceded areas prior to 1940.^25^ We compare these G2 children of forced migrants with the children of parents born in present-day Finland. In order to examine the intergenerational impact of forced migration, we vary the comparison group, which also serves as a means of controlling for bias due to observed and unobserved confounders. We compare the G2 children of two forced migrants with those whose parents were both born in the east of present-day Finland (the Eastern region in Figure 1) and those with both parents born in the rest of present-day Finland (the non-Eastern regions in Figure 1). We also follow prior research by making a more restrictive comparison between those whose parents were born in the neighboring municipalities on either side of the new border (i.e., both parents born on one side of the border in ceded Karelia vs. both parents born on the other side in present-day Finland, see eFigure S2; http://links.lww.com/EDE/C159 for spatial information regarding the border and eTable S5; http://links.lww.com/EDE/C159 for the coding of border areas based on municipalities).^30^ Taken together, this enables us to evaluate the intergenerational impact of forced migration.

### Data

We use population registers that cover the entire resident population of Finland from the end of 1970 until the end of 2020, in a total of 8,290,911 individuals. These registers allow us to identify people who were born in present-day Finland and in ceded Karelia by the municipality of birth. The data also allow us to link people across generations, if children and their parents were alive and residing in the same household at the end of 1970, or if children were born after 1970.

### Study Population

Due to our interest in adult mortality and the relatively young age of the G2 children of forced migrants, we study all adult men and women who can be observed from age of 17 years and were resident in Finland from 1970 to 2020, implying that the first cohort we study is children born in 1953 (a Lexis diagram for the study design is available in eFigure S1; http://links.lww.com/EDE/C159). The last cohort we study is children born in 1972, essentially because later-born cohorts very rarely have parents who were born in ceded Karelia. We drop individuals for whom both parents cannot be identified (7.6% of all Finnish-born individuals in the cohorts born 1953–1972). Otherwise, there are no missing data on any variables that are used in the analysis. The primary group of interest, G2 children of forced migrants, consists of persons born in Finland with both parents born in ceded Karelia (9454 men and 8925 women). These are compared with individuals with two parents born in Eastern Finland (122,556 men and 117,624 women) or in the rest of present-day Finland (369,915 men and 352,548 women). The more restrictive analysis compares individuals with two parents born in the municipalities of present-day Finland next to the new border (10,175 men and 9747 women) with individuals with two parents born on the other side of the border, in neighboring municipalities of ceded Karelia (2310 men and 2281 women). Sensitivity analysis based on alternative categorizations for children with parents born in other areas and the removal of children whose parents were born in Wyborg (the largest town in ceded Karelia) are available in the supplementary materials (eTables S1–S4; http://links.lww.com/EDE/C159).

### Variables and Statistical Analysis

We use semiparametric survival analysis, Cox proportional hazards models, to analyze all-cause and cause-specific mortality. Time of death refers to the calendar year of death. Cause of death is based on standard International Classification of Diseases (ICD) codes. For the cause-specific analysis, we group cause of death into established categories that relate to individual risk behavior (see eTable S6; http://links.lww.com/EDE/C159 for the coding of cause of death categories). People are right-censored at death, emigration, or at the end of 2020. We exclude those who lived abroad and returned to Finland during the study period, meaning that we study the stationary population. The exposure of interest and comparison groups are based on the parental municipality of birth for both parents. Control variables include birth year (categories for each year), region of residence at age of 17 years (20 categories), and mother’s and father’s educational level (categorized according to the International Standard Classification of Education 2011 classification as primary, upper-secondary, postsecondary nontertiary, short-cycle tertiary, bachelor’s or equivalent, master’s or equivalent, and doctoral or equivalent). For convenience, we present the results of our models using hazard ratios (HRs) and 95% confidence intervals (CIs). However, we note that all analysis is based on total population data, meaning that there is no uncertainty regarding design-based inferences. Sensitivity analysis that also incorporates children’s own educational level (categorized in the same way as for parents) and observes them from age of 35 years, instead of from age of 17 years, is available in the supplementary materials (eTables S1–S3; http://links.lww.com/EDE/C159). These sensitivity analyses also include results for individuals with one parent born in ceded Karelia and one born in Eastern Finland.

## RESULTS

Descriptive statistics and crude death rates for our study population are provided in Table 1 (with more detailed summary statistics provided in eTable S0; http://links.lww.com/EDE/C159). Prior to covariate adjustment, there are clear differences between the death rates of children of forced migrants, men and women aged 17–67 years with two parents born in ceded Karelia, and the other reference groups. In short, for both men and women, the crude death rate is highest for individuals with two parents born in ceded Karelia, followed by those with two parents born in Eastern Finland, and lowest for those with two parents born in the rest of present-day Finland.

**TABLE 1.** Descriptive Statistics and Crude Death Rates

Population	Person-Years	Deaths	Crude Rate (per 100,000 Person-Years)
Women 17–67			
Parents both born in ceded Karelia	358,110	543	152
Parents both born in ceded Karelia: border areas	90,905	125	140
Parents both born in Eastern Finland	4,509,404	5,875	130
Parents both born in Eastern Finland: border areas	382,261	502	130
Parents both born in the rest of present-day Finland	13,101,233	14,377	110
Men 17–67			
Parents both born in ceded Karelia	378,245	1,261	333
Parents both born in ceded Karelia: border areas	93,637	292	310
Parents both born in Eastern Finland	4,652,704	14,739	317
Parents both born in Eastern Finland: border areas	394,886	1,224	310
Parents both born in the rest of present-day Finland	13,628,386	34,021	250

**FIGURE 2.**
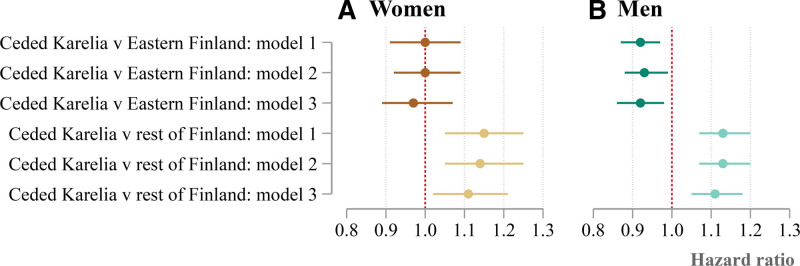
The all-cause mortality of women and men with both parents born in ceded Karelia, relative to different reference populations. The figure shows hazard ratios, and accompanying 95% confidence intervals, from semiparametric survival models of all-cause mortality. Hazard ratios in the first three rows (marked “Ceded Karelia v Eastern Finland”) are for women and men with two parents born in ceded Karelia relative to those with two parents born in Eastern Finland. The bottom three rows (marked “Ceded Karelia v rest of Finland”) are for women and men with two parents born in ceded Karelia relative to those with two parents born in the rest of present-day Finland (the Northern, Southern, and Western regions). Models 1–3 refer to alternative specifications of the survival model. Model 1 includes birth year. Model 2 includes birth year, father’s education, and mother’s education. Model 3 includes all the covariates in model 2 as well as the region of residence at age of 17 years.

The results from models controlling for observed confounders are shown in Figure 2. The all-cause mortality of children of forced migrants remains higher than children of parents born in the rest of present-day Finland (excluding the Eastern region). This is true for women and men and for all three model specifications. The most detailed specification (model 3) controls for year of birth, mother’s and father’s education level, and region of residence at age of 17 years. It generates estimated HRs of 1.11 for women (95% CI: 1.02, 1.21) and 1.11 for men (95% CI: 1.05, 1.18). There is almost no difference between female children of forced migrants and the female children of parents born in Eastern Finland. The same comparison for men shows that children of forced migrants have slightly lower mortality. For this comparison, model 3 gives HR of 0.97 for women (95% CI: 0.89, 1.07) and 0.92 for men (95% CI: 0.86, 0.98).

To further control for unobserved heterogeneity, Table 2 makes an additional comparison. It compares those with two parents born in the border municipalities of ceded Karelia versus those with two parents born in the border municipalities of Eastern Finland. Although this comparison has less statistical power, it can be argued that it comes closer to evaluating the intergenerational impact of forced migration, primarily due to the similarities (prior to the war) between individuals who are living in municipalities that are on either side of the border. For example, we expect these individuals to have similar levels of health and similar health behaviors, on average, as well as with respect to other potential confounders prior to the ceding of Karelia that are unobserved in our data. In this case, model 3 (which controls for the same variables as model 3 before) generates an HR of 0.90 for women (95% CI: 0.72, 1.11) and 0.96 for men (95% CI: 0.83, 1.10). We obtain similar results in our sensitivity analysis that includes those with parents born in municipalities divided by the new border and excludes those with parents born in Wyborg, which was one of the more economically developed towns in Finland prior to the war. We also run a range of other sensitivity analyses (see supplementary materials), which provide similar evidence to that shown in the main results. This includes results showing that those with two parents born in ceded Karelia either tend to exhibit an advantage, or negligible difference, as compared with those who have one parent born in ceded Karelia and one born in Eastern Finland.

**TABLE 2.** The All-Cause Mortality of Women and Men With Both Parents Born in Ceded Karelia, Relative to Different Reference Populations

	Ceded Karelia Versus Eastern Finland			
	HR	95% CI	Deaths	Person-years
Women				
Whole population				
Model 1	1.00	0.91, 1.09	6,418	4,867,513
Model 2	1.00	0.92, 1.09		
Model 3	0.97	0.89, 1.07		
Border municipalities				
Model 1	0.94	0.77, 1.14	627	473,165
Model 2	0.94	0.78, 1.15		
Model 3	0.90	0.72, 1.11		
Men				
Whole population				
Model 1	0.92	0.87, 0.97	16,000	5,030,949
Model 2	0.93	0.88, 0.99		
Model 3	0.92	0.86, 0.98		
Border municipalities				
Model 1	0.89	0.79, 1.01	1,516	488,523
Model 2	0.93	0.82, 1.05		
Model 3	0.96	0.83, 1.10		

The table shows hazard ratios, and accompanying 95% confidence intervals, from semiparametric survival models of all-cause mortality. Results are for two different comparisons: all those with two parents born in ceded Karelia versus all those with two parents born in Eastern Finland (whole population), and all those with two parents born in the border municipalities of ceded Karelia versus all those with two parents born in the border municipalities of Eastern Finland (border municipalities). Note that we exclude those municipalities that include(d) territory on both sides of the border. Model 1 includes birth year. Model 2 includes birth year, father’s education, and mother’s education. Model 3 includes all the covariates in model 2 as well as the region of residence at age 17.

**FIGURE 3.**
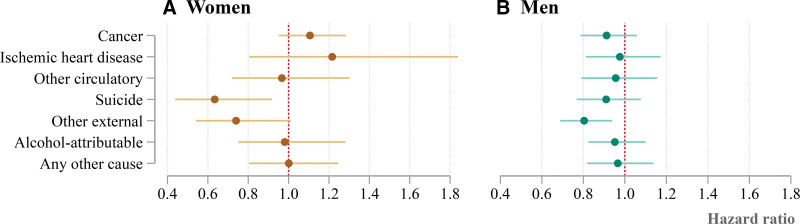
The cause-specific mortality of women and men with two parents born in ceded Karelia, relative to those with two parents born in Eastern Finland. The figure shows hazard ratios, and accompanying 95% confidence intervals, from semiparametric survival models of cause-specific mortality, adjusted for effects of birth year, father’s education, mother’s education, and region of residence at age of 17 years.

To expand upon these results, we carry out a similar analysis for cause-specific mortality (Figure 3 and Table 3). Compared with children of parents who were born in Eastern Finland, children of forced migrants have lower mortality with respect to suicide and other external causes of death. This remains the case in models that control for birth year, parental education, and region of residence at age of 17 years, where HRs for suicide are 0.63 for women (95% CI: 0.44, 0.92) and 0.91 for men (95% CI: 0.77, 1.08), and HRs for other external causes are 0.74 for women (95% CI: 0.54, 1.01) and 0.80 for men (95% CI: 0.69, 0.94). There are other manifest differences, but they are smaller than for suicides and other external causes and have less statistical power. The most notable excess mortality is from ischemic heart disease for the female children of forced migrants as compared to women with both parents born in Eastern Finland. When restricting the analysis to municipalities next to the border, there is also some evidence of excess alcohol-attributable mortality among the female children of forced migrants.

**TABLE 3.** The Cause-Specific Mortality of Women and Men Whose Parents Were Both Born in Ceded Karelia, Relative to Different Reference Populations

	All Ceded Karelia Versus All Eastern Finland				Border Municipalities Only			
	Model 1		Model 2		Model 1		Model 2	
	HR	95% CI	HR	95% CI	HR	95% CI	HR	95% CI
Women								
Cancer	1.07	0.93, 1.23	1.11	0.95, 1.28	1.12	0.83, 1.51	0.99	0.71, 1.38
Ischemic heart disease	1.26	0.86, 1.85	1.22	0.80, 1.84	1.04	0.42, 2.58	0.71	0.25, 2.01
Other circulatory	1.02	0.77, 1.34	0.97	0.72, 1.30	1.00	0.54, 1.85	0.87	0.44, 1.74
Suicide	0.70	0.49, 1.00	0.63	0.44, 0.92	0.58	0.26, 1.27	0.53	0.23, 1.24
Other external	0.79	0.59, 1.07	0.74	0.54, 1.01	0.32	0.13, 0.78	0.28	0.11, 0.71
Alcohol attributable	1.06	0.82, 1.36	0.98	0.75, 1.28	1.41	0.83, 2.38	1.50	0.82, 2.73
Any other cause	1.01	0.82, 1.24	1.00	0.80, 1.25	0.84	0.51, 1.38	1.07	0.62, 1.83
Men								
Cancer	0.98	0.85, 1.12	0.91	0.79, 1.06	0.89	0.65, 1.21	0.89	0.62, 1.26
Ischemic heart disease	0.93	0.78, 1.10	0.98	0.81, 1.17	1.00	0.69, 1.47	1.14	0.74, 1.77
Other circulatory	1.01	0.85, 1.21	0.96	0.79, 1.16	0.96	0.65, 1.42	1.02	0.65, 1.60
Suicide	0.83	0.71, 0.97	0.91	0.77, 1.08	0.82	0.58, 1.14	0.99	0.68, 1.43
Other external	0.78	0.68, 0.91	0.80	0.69, 0.94	0.87	0.64, 1.18	0.95	0.67, 1.33
Alcohol attributable	0.98	0.86, 1.12	0.95	0.83, 1.10	1.04	0.77, 1.41	1.08	0.77, 1.52
Any other cause	0.99	0.85, 1.15	0.97	0.82, 1.14	0.74	0.50, 1.07	0.74	0.49, 1.12

The table shows hazard ratios, and accompanying 95% confidence intervals, from semiparametric survival models of cause-specific mortality. Results are for two different comparisons: (1) all those with two parents born in ceded Karelia versus all those with two parents born in Eastern Finland, and (2) all those with two parents born in the border municipalities of ceded Karelia versus all those with two parents born in the border municipalities of Eastern Finland (border municipalities only). Note that we exclude those municipalities that include(d) territory on both sides of the border. Model 1 includes birth year. Model 2 includes birth year, father’s education, mother’s education, and region of residence at age of 17 years.

## DISCUSSION

In this study, we examined all-cause and cause-specific adult mortality among the children of forced migrants. We capitalized on a unique case of forced migration, from ceded Karelia to present-day Finland, which essentially eliminated selection as one of the primary explanations for the unique mortality patterns of G2 children of migrants. We aimed to establish whether or not the children of Karelian forced migrants experience elevated mortality compared with a population of present-day Finland whose parents were not forced to migrate. Contrary to prior research showing that the children of immigrants exhibit excess mortality, we find that this is not the case for children of forced migrants in a context where their parents were not selected for migration. Children with two parents born in ceded Karelia either have similar levels of mortality or lower mortality, as compared with children who have two parents born in the areas of present-day Finland next to ceded Karelia. In the absence of any potential influence of parental selection effects, this suggests that the children of immigrants are more likely to experience a mortality advantage, rather than any disadvantage.

Our findings imply that selection is central to explaining the mortality disadvantages that have been observed among the G2 in other high-income contexts (typically for those whose parents were born in low- and middle-income countries).^3–11^ Selection includes many aspects of life that determine who migrates, such as education, health, or family ties. Nevertheless, these are distinct from the impact of parental (forced) migration as an event that has potential intergenerational consequences for health. We estimate that there is no negative intergenerational impact of migration, net of selection, at least in this context.

The discussion of context naturally raises the issue of generalizability. We have noted that our study concerns a specific case of forced displacement, not only because an entire population was displaced but also because there were negligible differences between ceded Karelia and the rest of Finland, for example, in terms of culture, language, and society. This means that our findings may not be replicated in other contexts, in part because selection may play a more material role in determining the mortality of the G2 (as opposed to here, where it is largely absent), and in part because other contexts may vary with respect to the differences between origin and destination. In this respect, the forced migration in our context may be more similar to internal displacement than forced migration across international borders.

Origin and destination differences may explain the mortality of G2 children of migrants due to the extent to which adaptation is required in order to adjust to life in a new destination.^12,16^ Adaptation and the factors that determine adaptation, such as culture, discrimination, and institutional knowledge, are mechanisms that are unlikely to play a role in our context. Even though forced migrants from ceded Karelia experienced a loss of resources and suffered a disruption to their families, communities, and their lived experience,^29^ they have also been found in prior research to have adapted to life in their new homes within a short period of years.^31^ Nevertheless, adaptation may certainly play a material role in other contexts, for example in explaining the elevated mortality among children of immigrants across Europe.^12^

A question remains about the mechanisms that are most likely to explain our findings. Based on a recent review of the literature, the main factors that determine G2 mortality can be summarized as: (1) integration, (2) cultural/religious practices, (3) racism and discrimination, (4) healthcare, (5) parental demographic behavior, (6) parental health, (7) the social determinants of health, and (8) parental migration.^12^ We our trying to estimate the impact of the latter, and the first four are less likely to be important mechanisms in our context due to similarities between the origin and destination. By a process of elimination, we might assume that in our case, migration has a beneficial intergenerational impact in reducing G2 mortality due to its impact on G1 demographic behavior (e.g., age at birth), health (e.g., lowering stress), and the social determinants of health for G1 and G2 (e.g., increased opportunities for education and employment). We therefore recommend that future research tries to examine the role of these mechanisms in more detail. Here we note there is evidence from prior research that female forced migrants had fewer children^25^ and that male forced migrants experienced an increase in income over the long run,^32^ which suggests that these could be potential mechanisms that may explain our results.

With respect to cause of death, we find a mortality advantage that is largest for external causes of death, for both women and men. As for all-cause mortality, this advantage persists after controlling for various socioeconomic factors. It was beyond the scope of our study to establish why the children of forced migrants have lower mortality risks. However, our analysis suggests that the forced migration of parents may have a protective effect on their children via mechanisms that determine external causes, for example, mental health or the practice of risky behaviors. It would therefore be useful for future research to examine these mechanisms, focusing on suicide and other external causes, which are the causes in which lower mortality is most clearly evident.

Our findings also highlight the importance of making appropriate comparisons when seeking to evaluate the impact of forced migration on immigrants or their children. Eastern Finland is a more appropriate counterfactual for ceded Karelia when evaluating the impact of forced migration because the two areas were far more similar prior to the Second World War. We show that a comparison with other areas of present-day Finland produces very different results. It is also reassuring that our results are similar when we compare municipalities next to the border and in various sensitivity analyses (see eTables S1–S4; http://links.lww.com/EDE/C159).

A strength of our study is that we use individual-level data on the whole population, including all children of forced migrants (who are residents in Finland), and linkages to data on their parents. We go beyond prior research on the mortality of children of immigrants to examine cause-specific mortality, in addition to all-cause mortality. However, despite the ability of our study design to isolate the intergenerational impact of forced migration, it does have several weaknesses. Various potential confounders are unobserved, including the health of parents prior to forced migration. This is unlikely to bias our findings, particularly in the border analysis if, as we assume, health is similar between those living on either side of the border that did not exist prior to the forced migration. However, this remains an assumption, and it may be that our findings are biased to some extent by unobserved confounding. We also include only those people who have been resident in Finland since 1970, therefore excluding those who have emigrated prior to that year (although we anticipate that the number of people is small and would not have much impact on our results). In addition, it may be that our results do not generalize beyond our study population, including to other contexts. We have focused on adult mortality, and yet patterns may be different for child mortality among the children of immigrants.

## CONCLUSION

Our findings suggest that, if anything, forced migration has a beneficial impact on the adult mortality of subsequent generations, at least in the case where forced migrants are able to migrate to contextually similar locations that offer opportunities for rapid integration and social mobility. Further research is required to examine whether our results generalize to other migration contexts and to isolate the specific mechanisms that may explain the mortality disadvantage that is experienced by many children of immigrants in other contexts.

## Supplementary Material

## References

[1] Eurostat. First and Second-Generation Immigrants - a Statistical Overview - Statistics Explained. 2016. Available at: https://ec.europa.eu/eurostat/statistics-explained/index.php/First_and_second-generation_immigrants_-_a_statistical_overview. Accessed 27 July 2019.

[2] Eurostat. Foreign-Born People and Their Descendants - Main Characteristics. 2023. Available at: https://ec.europa.eu/eurostat/statistics-explained/index.php?title=Foreign-born_people_and_their_descendants_-_main_characteristics. Accessed 3 January 2023.

[3] Wallace M. Adult mortality among the descendants of immigrants in England and Wales: does a migrant mortality advantage persist beyond the first generation? J Ethn Migr Stud. 2016;42:1558–1577.

[4] Guillot M Khlat M Wallace M. Adult mortality among second-generation immigrants in France: Results from a nationally representative record linkage study. Demogr Res. 2019;40:1603–1644. 33986627 10.4054/demres.2019.40.54 PMC8114944

[5] Wallace M. Mortality advantage reversed: the causes of death driving all-cause mortality differentials between immigrants, the descendants of immigrants and ancestral natives in Sweden, 1997–2016. Eur J Popul. 2022;38:1213–1241 36507238 10.1007/s10680-022-09637-0 PMC9727037

[6] Khlat M Wallace M Guillot M. Divergent mortality patterns for second generation men of North-African and South-European origin in France: role of labour force participation. SSM Popul Health. 2019;9:100447. 31497637 10.1016/j.ssmph.2019.100447 PMC6718938

[7] Vandenheede H Willaert D Grande HD Simoens S Vanroelen C. Mortality in adult immigrants in the 2000s in Belgium: a test of the “healthy-migrant” and the “migration-as-rapid-health-transition” hypotheses. Trop Med Int Health. 2015;20:1832–1845. 26426523 10.1111/tmi.12610

[8] De Grande H Vandenheede H Gadeyne S Deboosere P. Health status and mortality rates of adolescents and young adults in the Brussels-Capital Region: differences according to region of origin and migration history. Ethn Health. 2014;19:122–143. 23438237 10.1080/13557858.2013.771149

[9] Manhica H Toivanen S Hjern A Rostila M. Mortality in adult offspring of immigrants: a Swedish national cohort study. PLoS One. 2015;10:e0116999. 25706297 10.1371/journal.pone.0116999 PMC4338186

[10] Bodewes AJ Agyemang C Kunst AE. All-cause mortality among three generations of Moluccans in the Netherlands. Eur J Public Health. 2019;29:463–467. 30544210 10.1093/eurpub/cky255

[11] Ho L Bos V Kunst AE. Differences in cause-of-death patterns between the native Dutch and persons of Indonesian descent in the Netherlands. Am J Public Health. 2007;97:1616–1618. 17666706 10.2105/AJPH.2006.086314 PMC1963302

[12] Wallace M Hiam L Aldridge R. Elevated mortality among the second-generation (children of migrants) in Europe: what is going wrong? A review. Br Med Bull. 2023;148:5–21. 37933157 10.1093/bmb/ldad027 PMC10724460

[13] Aldridge RW Nellums LB Bartlett S. Global patterns of mortality in international migrants: a systematic review and meta-analysis. Lancet. 2018;392:2553–2566. 30528484 10.1016/S0140-6736(18)32781-8 PMC6294735

[14] Guillot M Khlat M Elo I Solignac M Wallace M. Understanding age variations in the migrant mortality advantage: an international comparative perspective. PLoS One. 2018;13:e0199669. 29958274 10.1371/journal.pone.0199669 PMC6025872

[15] Spallek J Zeeb H Razum O. What do we have to know from migrants’ past exposures to understand their health status? A life course approach. Emerg Themes Epidemiol. 2011;8:6. 21843354 10.1186/1742-7622-8-6 PMC3169503

[16] Drouhot LG Nee V. Assimilation and the second generation in Europe and America: blending and segregating social dynamics between immigrants and natives. Annu Rev Sociol. 2019;45:177–199.

[17] Castles S. Towards a sociology of forced migration and social transformation. Sociology. 2003;37:13–34.

[18] IOM. Key Migration Terms. International Organization for Migration; 2024. Available at: https://www.iom.int/key-migration-terms. Accessed 15 February 2024.

[19] Bakewell O. Research beyond the categories: the importance of policy irrelevant research into forced migration. J Refug Stud. 2008;21:432–453.

[20] Stepputat F Sørensen NN. Sociology and forced migration. In: Fiddian-Qasmiyeh E Loescher G Long K Sigona N, eds. The Oxford Handbook of Refugee and Forced Migration Studies. Oxford University Press; 2014. Available at: http://www.oxfordhandbooks.com/view/10.1093/oxfordhb/9780199652433.001.0001/oxfordhb-9780199652433-e-010. Accessed 26 June 2019.

[21] Reed H Haaga J Keely CB. Demography of Forced Migration: Summary of a Workshop. National Academies Press; 1998. Available at: http://public.eblib.com/choice/publicfullrecord.aspx?p=3375768. Accessed 3 January 2017. 25057553

[22] Haukka J Suvisaari J Sarvimäki M Martikainen P. The impact of forced migration on mortality: a cohort study of 242,075 Finns from 1939–2010. Epidemiology. 2017;28:587–593. 28368943 10.1097/EDE.0000000000000669

[23] Saarela J Finnäs F. Forced migration and mortality in the very long term: did perestroika affect death rates also in Finland? Demography. 2009;46:575–587. 19771945 10.1353/dem.0.0069 PMC2831340

[24] Saarela J Elo IT. Forced migration in childhood: are there long-term health effects? SSM Popul Health. 2016;2:813–823. 28713854 10.1016/j.ssmph.2016.10.012 PMC5505672

[25] Saarela J Wilson B. Forced migration and the childbearing of women and men: a disruption of the tempo and quantum of fertility? Demography. 2022;59:707–729. 35322268 10.1215/00703370-9828869

[26] Heudtlass P Speybroeck N Guha-Sapir D. Monitoring mortality in forced migrants—can Bayesian methods help us to do better with the (Little) data we have? PLoS Med. 2015;12:e1001887. 26485006 10.1371/journal.pmed.1001887 PMC4617888

[27] Bauer TK Giesecke M Janisch LM. The impact of forced migration on mortality: evidence from German pension insurance records. Demography. 2019;56:25–47. 30499060 10.1007/s13524-018-0742-z

[28] Singh K Karunakara U Burnham G Hill K. Forced migration and under-five mortality: a comparison of refugees and hosts in North-western Uganda and Southern Sudan. Eur J Popul/Revue européenne de Démographie. 2005;21:247–270.

[29] Kuusisto-Arponen A-K. The mobilities of forced displacement: commemorating Karelian evacuation in Finland. Soc Cult Geogr. 2009;10:545–563.

[30] Becker SO Grosfeld I Grosjean P Voigtländer N Zhuravskaya E. Forced migration and human capital: evidence from Post-WWII population transfers. Amer Econ Rev. 2020;110:1430–1463.

[31] Waris H Jyrkilä V Raitasuo K Siipi J. Siirtoväen sopeutuminen: Tutkimus Suomen karjalaisen siirtoväen sosiaalisesta sopeutumisesta. Otava; 1952.

[32] Sarvimäki M Uusitalo R Jäntti M. Long-Term Effects of Forced Migration. IZA Discussion Paper Series; 2009:46.

